# Reduction Expansion Synthesis as Strategy to Control Nitrogen Doping Level and Surface Area in Graphene

**DOI:** 10.3390/ma8105359

**Published:** 2015-10-16

**Authors:** Russell Canty, Edwin Gonzalez, Caleb MacDonald, Sebastian Osswald, Hugo Zea, Claudia C. Luhrs

**Affiliations:** 1 Mechanical and Aerospace Engineering Department, Naval Postgraduate School, Monterey, CA 93943, USA; cantyrs@illinois.navy.mil (R.C.); edwin7gonzalez@gmail.com (E.G.); caleb.macdonald012@gmail.com (C.M.); 2 School of Materials Engineering, Purdue University, West Lafayette, IN 47907-2045, USA; sosswald@purdue.edu; 3 Departamento de Ingeniería Química y Ambiental, Universidad Nacional de Colombia, Bogotá, 111321, Colombia; hrzear@unal.edu.co

**Keywords:** reduction-expansion-synthesis, nitrogen-doped graphene

## Abstract

Graphene sheets doped with nitrogen were produced by the reduction-expansion (RES) method utilizing graphite oxide (GO) and urea as precursor materials. The simultaneous graphene generation and nitrogen insertion reactions are based on the fact that urea decomposes upon heating to release reducing gases. The volatile byproducts perform two primary functions: (i) promoting the reduction of the GO and (ii) providing the nitrogen to be inserted *in situ* as the graphene structure is created. Samples with diverse urea/GO mass ratios were treated at 800 °C in inert atmosphere to generate graphene with diverse microstructural characteristics and levels of nitrogen doping. Scanning electron microscopy (SEM) and transmission electron microscopy (TEM) were used to study the microstructural features of the products. The effects of doping on the samples structure and surface area were studied by X-ray diffraction (XRD), Raman Spectroscopy, and Brunauer Emmet Teller (BET). The GO and urea decomposition-reduction process as well as nitrogen-doped graphene stability were studied by thermogravimetric analysis (TGA) coupled with mass spectroscopy (MS) analysis of the evolved gases. Results show that the proposed method offers a high level of control over the amount of nitrogen inserted in the graphene and may be used alternatively to control its surface area. To demonstrate the practical relevance of these findings, as-produced samples were used as electrodes in supercapacitor and battery devices and compared with conventional, thermally exfoliated graphene.

## 1. Introduction

It is well known that the methods used to fabricate and process materials largely determine the characteristics of the resulting products and are therefore key to achieve the desired properties and performance. Graphene, the two dimensional material consisting of an atom thick layer of carbon in a sp^2^ hexagonal arrangement [1], and its derivatives, are no exception. In recent years, the number of reports attempting to tailor graphene properties has grown at stunning rates [2]. One of the common routes used to generate graphene uses graphite oxide (GO) as feedstock [3,4]. GO can be easily generated from the oxidative treatment of graphite flakes [5,6]. Once the oxygen groups provided by these procedures have been attached to the graphite structure to generate GO, its transformation to graphene can be performed by various reducing methods, including thermal annealing [3], plasma treatment [7], sonication [8,9], electrochemical routes [10] and use of multiple reducing agents [11,12,13,14,15]. This manuscript describes the use of GO along with a reducing expansion agent, to produce doped graphene in which the amount of nitrogen, or alternatively the surface area, can be controlled. 

While graphene produced from GO has been reported as a material with a promising electrochemical performance when used for the manufacture of supercapacitor and lithium ion battery electrodes, its practical energy storage capacity is still well below the theoretical values [16,17]. There are, however, strategies to improve electrochemical properties and specific capacitance/capacity of graphene, including the incorporation of heteroatoms (e.g., N, B and O), which are known to be electron donors capable to modify the electronic structure of graphene [18,19,20,21]. Several doping methods were found to successfully incorporate nitrogen atoms into graphene, including chemical vapor deposition [20,22,23], arc discharge, nitrogen plasma [24,25], and thermal treatments [14,26,27]. Unfortunately, the majority of these methods suffer from high cost, low yield, and/or use of toxic precursors, or involve sophisticated equipment that makes scalability impractical. Our group recently introduced the reduction expansion synthesis (RES) using urea as an inexpensive, simple, and easily scalable process to generate nitrogen doped graphene in a single step [12,28,29,30]. The method was previously employed to produce highly divided reduced metals [31,32,33].

The intial work, published in 2010 [12], was followed by multiple studies from other authors using urea as reducing agent [34,35,36,37,38]. However, to the best of our knowledge, these reports describe the levels of doping being dependant only on temperature or processing time. With the patent describing the method recently issued [39], it became neccessary to further investigate the effects of urea addition on the properties of the resulting graphene. Here, we provide evidence that the amount of urea in the precursor mixture has a profound effect on the structure and composition of the produced graphene, and may be used to control the level of doping or specific surface area of the material. We finally demonstrate the importance of these findings by reporting the performance of the as-produced graphene as both supercapacitor and lithium ion battery electrodes.

## 2. Results and Discussion

**Figure 1:**
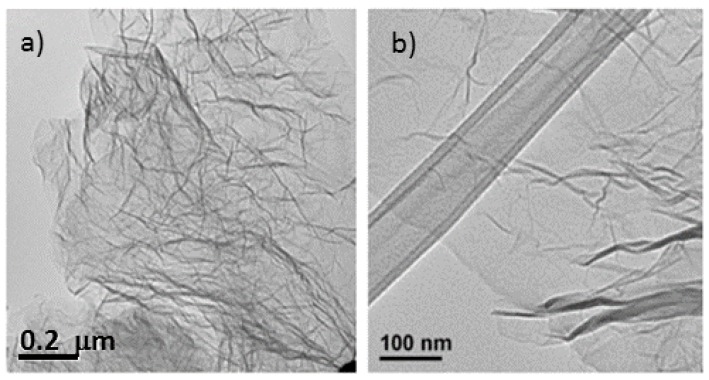
Transmission electron microscopy (TEM) image of the same un-doped sample. (**a**) Exhibiting entangled graphene sheets; (**b**) TEM of doped graphene sheet (precursor had a mass ratio urea/graphite oxide (GO) of 0.25) displaying a wrinkled sheet.

**Figure 2:**
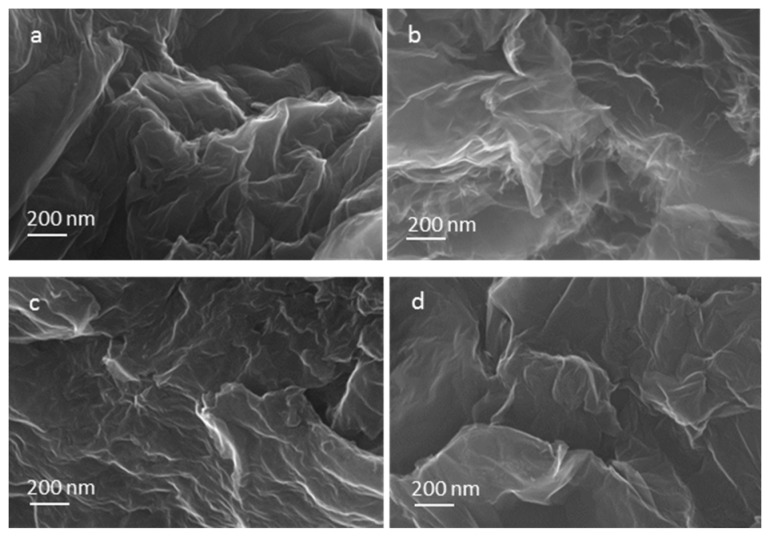
Scanning electron microscopy (SEM) images of samples with diverse doping levels. (**a**) Thermally exfoliated Graphene; (**b**) Graphene produced from precursor with mass ratio urea/GO = 0.6; (**c**) mass ratio urea/GO = 0.8 and (**d**) mass ratio urea/GO = 1.

The microstructural characteristics of the nitrogen-doped graphene samples were analyzed by scanning electron microscopy (SEM) and transmission electron microscopy (TEM) (Figure 1 and Figure 2) and compared with thermally exfoliated samples (no urea). The dispersion of the reduced graphene specimens in a solvent using sonication, as preparation step for TEM sample analysis, renders disordered sheets that entangle with each other (Figure 1). Higher magnification images reveal to be 1–5 atomic layer sheets. The SEM micrographs showed that more than producing a set of individual layers with different orientations, the structure remains attached in certain sections, creating a multilayered architecture similar to a honeycomb. The SEM images of samples with diverse levels of doping (Figure 2) indicate a more compact structure for specimens produced with higher amounts of urea such as mass ratio urea/GO = 0.8 or 1.

The first evidence of nitrogen insertion in the doped samples was encountered in the spectroscopic data generated. The energy dispersive X-ray spectroscopy (EDS) spectra (Supplementary Materials Figure S1) of samples prepared with urea present a clear nitrogen peak located between the carbon and oxygen peaks, near 0.4 keV, while the specimen reduced by simple thermal exfoliation lacked such a feature. Previous studies commented on the amounts of oxygen encountered in thermally reduced samples; the GO loses most of the oxygen functional groups but the conversion does not reach a 100%. Some studies [7,12] estimate that thermal exfoliation routes alone render 10%–11% oxygen in the reduced graphene final structure, making the appearance of a small oxygen peak in the EDS spectra, as the ones observed in this study, an expected outcome. When the samples were analyzed by energy electron loss spectroscopy (EELS), the presence of nitrogen was confirmed by the appearance of additional peaks above 400 eV, along the characteristic graphitic 1s-π* and 1s-σ* peaks in the K-edge region at 285 and 291 eV, respectively reported by other studies [40,41]. 

**Figure 3:**
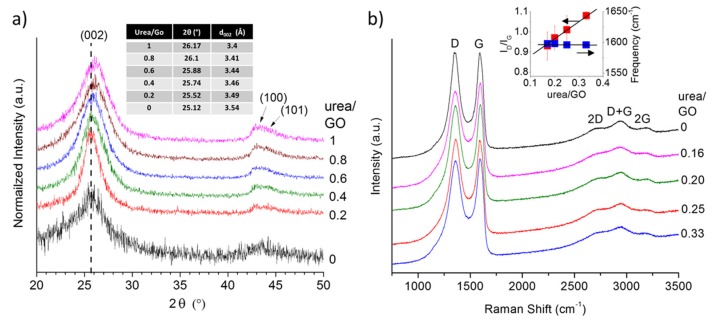
X-ray diffraction (XRD) pattern showing a shift in the *d*_002_ peak toward higher angles as the urea/GO ratio in the precursor increases (**a**) and Raman spectra (**b**) of urea/GO-derived samples in comparison to thermally exfoliated graphene. The Raman intensity ratio between D and G band, *I*_D_*/I*_G_, increases with increasing urea content while the G band position remains nearly unchanged (right insert).

To obtain further insight into potential structural changes in the graphene with increasing urea content, we analyzed all samples using X-ray diffraction (XRD). The obtained XRD patterns (Figure 3a) reveal a shift in the *d*_002_ peak toward higher angles (smaller interlayer spacing) as the urea/GO ratio in the precursor increases. This suggest that the presence of urea not only affects the exfoliation process, but that the amount of urea may be used to control the level of exfoliation and thus the interlayer spacing in graphene samples. As shown previously, the decomposition of urea generates additional volatile species that aid the reduction of the GO (more complete reduction), but also results in an overall less effective expansion process [28]. Consequently, with increasing urea/GO mass ratio, the level of exfoliation decreases and the interlayer spacing is approaching that of graphite. This effect has been observed before in other nitrogen doped graphene samples; prior work has hypothesized that a reduction in the interlayer spacing, as nitrogen is introduced into the graphene structure, might be due to ammonia corrosive processes [42] or to the sheet defective structure [43]. However, given the different nitrogen species encountered when doping graphene (in graphitic, pyridinic, and pyrrolic configurations) it is also conceivable that an uneven distribution of charges in the different sheets promote the creation of attractive forces that increase their interactions. This phenomenon is worth further investigation and will be the subject of attention in future research. In addition, it can be noted that the relative intensity of the (100)/(101) features also increases with increasing urea content, resulting in a more complete reduction and a smaller number of in-plane defects. At the same time, other byproducts of the urea decomposition (e.g., ammonia) react with the graphene, leading to the incorporation of nitrogen atoms into the carbon lattice (doping), as discussed in detail in a previous studies [12,34,36,37].

**Figure 4:**
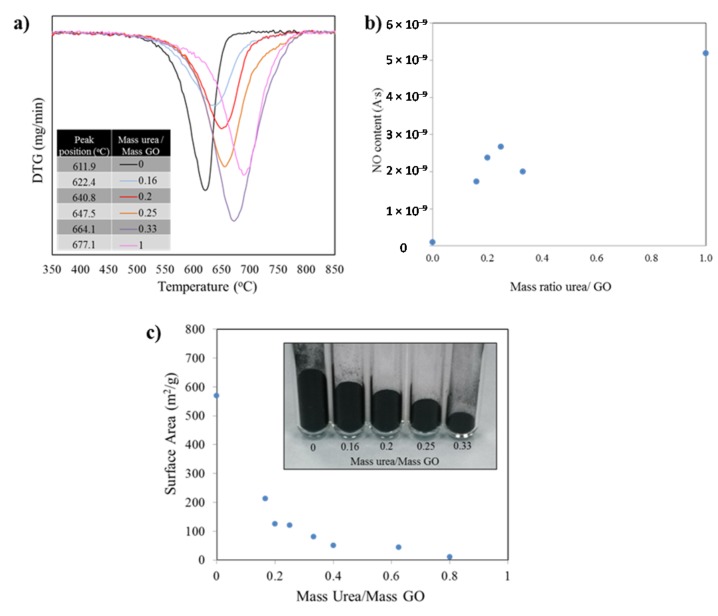
(**a**) Derivative (DTG) curve of the weight loss observed during the samples burn off process shifts to higher temperatures as the amounts of nitrogen introduced in the structures increase; (**b**) Corresponding mass spectroscopy data recorded during burn-off (mass 30). The measured ion current was normalized to the sample weight to account for differences in sample mass; (**c**) Variation in surface area values as the mass ratio of urea/GO increase. The same mass of sample for diverse formulations show a significant difference in mass volume.

Figure 3b depicts the first and second order Raman spectra of the urea/GO-derived graphene samples, in comparison to thermally exfoliated graphene. All spectra exhibit the characteristic defect-induced D band and graphitic G band of sp^2^-carbons, as well as the resulting second order and combination modes. The most notable spectral change is the increase in the D band-to-G band intensity ratio (*I*_D_*/I*_G_) with increasing urea content. Although, according to XRD data (Figure 4), both in-plane and stacking order increase with higher urea content, the number of nitrogen atoms incorporated into the carbon lattice also rises. The nitrogen atoms are defects within the graphene lattice (substitutable impurities), and consequently, contribute to the D band intensity, yielding the observed increase in *I*_D_/*I*_G_. The *I*_D_/*I*_G_ value of the thermally exfoliated sample is higher (~1.06) than most urea/GO-derived samples, even without any nitrogen inclusions. This discrepancy results from differences in the exfoliation mechanism as previously discussed [28]. Given this sensitivity to the presence of nitrogen impurities, the *I*_D_/*I*_G_ therefore provides a fast and convenient tool to estimate the concentration of nitrogen atoms in doped-graphene samples. For a more detailed description of the Raman spectra of urea/GO-derived graphene samples, the reader is referred to work by Mowry *et al.* [28]. 

While Raman measurements provided indirect evidence for a direct relationship between urea content and level of N-doping, we conducted thermogravimetric analysis (TGA) coupled with mass spectroscopy (MS) to directly evaluate the N-content of the samples. TGA was also conducted in order to identify the differences in thermal stability of the diverse samples. The burn off temperatures of the specimens were studied under an oxygen-containing atmosphere and the mass spectral signal of the byproducts generated as the carbon structure burns off. Figure 4a presents the first derivative of the mass as a function of the temperature. The minima indicate the temperatures at which the highest mass loss occurred. Such temperature value for each peak is listed in the inset table for the figure and further demonstrates the dramatic shift in thermal stability. The burn off process for the sample with no nitrogen, thermally reduced graphene (no urea in precursor), yielded a mass loss peak temperature of 611.9 °C. The addition of nitrogen to the graphene structure shifts the position of the mass loss derivative, such that, for a sample prepared from a urea/GO 1/1 ratio the peak position is 677.1 °C, providing evidence that the thermal stability increases for the doped material. It is important to note that the oxidation of all samples starts in a similar temperature range around 475–520 °C. However, the rate of oxidation (slope of curve) at the early stages of the burn-off and the total temperature window in which the oxidation takes place (width of the curve) vary substantially. 

The TGA-MS results are thus in good agreement with XRD and Raman data, providing further evidence for the increment in structural order with increasing urea content. While all samples consist of graphitic sp^2^ (hence similar onset temperatures for oxidation), the burn off rate is higher for samples containing less urea. These samples are more exfoliated, and thus more porous, as compared to graphene with higher stacking order. At temperatures around 500 °C, mass transport becomes the rate-limiting reaction step in carbon nanomaterial oxidation, leading to reduced oxidation rate in low porosity materials. In addition, the lower concentration of two dimensional defects (due to nitrogen repair of lattice defects during exfoliation with urea in a nitrogen atmosphere) also contributes to the higher thermal stability with increasing urea content. 

Mass spectra analyses of gasses generated during TGA runs revealed the formation of CO_2_ as main byproduct for pure graphene and CO_2_ and NO for doped graphene under the atmosphere and conditions used. As concentrations of nitrogen in the precursor increase, the temperature at which maximum NO and CO_2_ leave the structure/off gas increases (Figure 4a). The mass spectroscopy data recorded during burn-off for NO (mass 30), that is, the measured ion current normalized to the sample weight to account for differences in sample mass, follow an upward trend as the amount of urea in the samples increase. With the exception of one of the points, the rest of values confirmed the larger amounts of nitrogen in the samples as the urea/GO mass ratio increase.

The Brunauer Emmet Teller (BET) surface area measurements for samples with diverse doping levels show an exponential decay as the amount of urea used in the precursor increase (correlated to the nitrogen doping level). Such change is also evident when weighing the same amount of the different powders: As the amount on nitrogen introduced increase, the level of exfoliation decreases. See Figure 4c inset. After a value of mass ratio urea/ GO of 0.6 is reached, the surface area collapses to values near to that of untreated graphite. It is worth noting that by modifying the amount of urea in the precursor mix, the surface area value in the resulting product can be controlled. Measurements were conducted multiple times to corroborate repeatability, in all cases falling within a 5% of the expected values.

**Figure 5:**
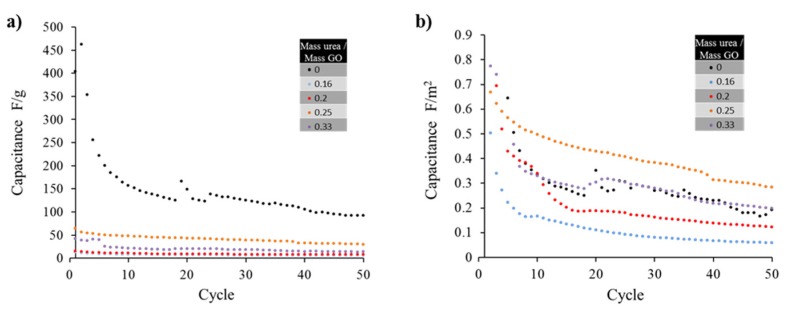
(**a**) The capacitance values of the samples drastically decrease after the first 10 cycles, only the sample with un-doped graphene (mass ratio urea/GO = 0) seems to maintain high capacitance (*ca.* above 100 F/g); (**b**) Capacitance values normalized as F/m^2^ show that the nitrogen doping actually improves the cycle life when samples surface area is considered, reaching a maximum for the sample prepared with an urea/GO mass ratio of 0.25.

For the supercapacitor tests, the specific capacitance of some of the samples are plotted in Figure 5a, revealing a maximum value for thermally exfoliated graphene (no urea) when measured in F/g. This result can be related to the high surface area of the material yielding a similarly high specific capacitance. After 50 cycles the capacitance for the pure graphene, that started at a value of 400 F/g, approached 127 F/g. The latter value is consistent with results (117 F/g) obtained by other groups [4,39,40,41,42]. The large reduction in surface area between un-doped and doped graphene dominates the capacitance, as the lowest doping concentration resulted in a threefold decrease in surface area and a similar reduction in capacitance. The reduction in capacitance ranged from a 70 % decrease to 90 % in specific capacitance for ratios 0.25 and 0.16, respectively. When the data is transformed to normalize surface area values, using F/m^2^ (Figure 5b) instead of F/g units (Figure 5a), an optimal doping for the sample generated from mass ratio urea/GO precursor of 0.25 is identified. The later shows a 50 % more specific capacitance than sample 0.33 and 300 % more specific capacitance than sample with ratio of 0.2. That is, for similar values of surface area, the doped sample which precursor was made from urea/GO ratios of 0.25 outperforms the thermally exfoliated graphene sample (no urea added).

The sample identified as 0.25 (mass ratio urea/GO precursor) demonstrated superior capacitive characteristics to the un-doped graphene proving that the doping has a positive benefit to the electrical properties of the material. The 0.33 sample performed nearly identically to the bare thermally exfoliated graphene. The 0.2 and 0.16 samples demonstrated worse electrical characteristics than others, further emphasizing the presence of an optimal peak doping concentration in the vicinity of 0.25.

Nitrogen-doped graphene has also been reported to provide superior electrochemical performance and cycle stability in Li-ion batteries, as compared to pristine graphene [44,45]. The enhanced Li uptake has been ascribed to a larger number of defects and increased disorder as well as the higher electronegativity of N-doped graphene. 

**Figure 6:**
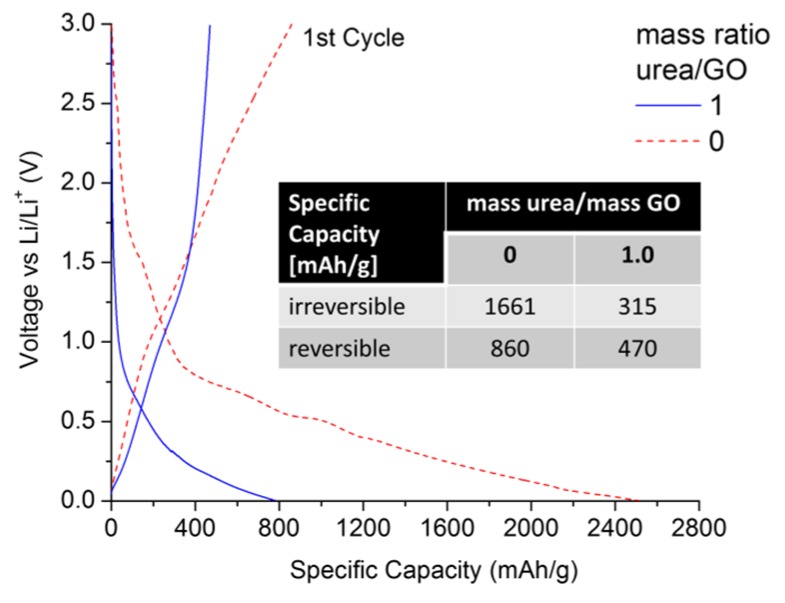
Lithiation/delithiation voltage profiles of urea/GO-derived graphene (mass ratio of 1.0) in comparison to thermally exfoliated graphene. All data were recorded in half-cell configuration (*vs.* Li/Li^+^) using a current of C/10.

Figure 6 compares the first lithiation/delithiation cycle of an urea/GO-derived (mass ratio = 1) sample with that of thermally exfoliated graphene, using a C-rate of C/10 (with 1 C = 372 mA/g).

As expected, the thermally exfoliated graphene is subject to a high irreversible capacity loss (>1600 mAh/g), exhibiting a reversible specific capacity of ~860 mAh/g at the end of the 1st cycle. However, in contrast to previous studies [44,45], the N-doped graphene showed lower irreversible and reversible capacities of 315 and 470 mAh/g, respectively. This is an important finding for several reasons. (1) Unlike in early studies, N-doping via thermal exfoliation of urea-/GO mixtures yields samples with higher structural order and less specific surface area. Therefore, the effects of nitrogen defects and structural disorder on overall Li uptake can be decoupled; (2) Surface area and nitrogen content can be controlled by adjusting the urea/GO mass ratio. This is important, as from a practical point of view, high reversible capacities are not necessarily favorable if they are linked to very high irreversible 1st cycle capacities, which raises challenges in cell manufacturing and anode/cathode balancing. More favorable are materials that exceed the practical specific capacity of graphite (~330 mAh/g), but also maximize the C_reversible_/C_irreversible_ capacity ratio. Under this consideration, N-doped graphene would be the more favorable anode material (0.52 *vs.* 1.49); (3) Given the lower surface area values of N-doped graphene (<10 m^2^/g), we can conclude that the addition of urea is an effective way to control the extent of GO exfoliation, allowing for the optimization of C_reversible_/C_irreversible_ in graphene materials. 

The resulting capacitance of the un-doped graphene prepared by thermal exfoliation in units of F/g was remarkably higher when compared with doped samples. Specific capacitance has been strongly correlated to the surface area of the graphene or doped graphene employed in the electrode material. Such phenomenon could be explained when relating the capacitance to the number of charges interacting at the electric double layer: the higher the surface area, the larger the sites where charges in the interface could be created. However, once the capacitance values get normalized by surface area, using F/m^2^ units, there seems to be an optimal value (sample with 0.25), demonstrating that despite the reduction in surface area, there must be an addition in charge mobility that improves sample performance.

Electrochemical testing demonstrated that addition of urea offers a unique pathway to optimize the critical C_reversible_/C_irreversible_ ratio in lithium ion batteries. High control over surface area and interlayer spacing may also provide opportunities for other advanced battery technologies, such as sodium ion batteries, where conventional graphitic materials are unsuitable. 

While an in-depth discussion of the electrochemical performance of urea/GO-derived graphene is outside the scope of this study and will be published elsewhere, the presented data demonstrates the unique characteristics of urea/GO-derived graphene and the opportunities in energy storage applications. 

## 3. Experimental Section

### 3.1. Materials and Methods

GO was produced from synthetic graphite powder (particle size < 20 μm, Aldrich, St. Louis, MO, USA) following a variation of the Hummer’s method [46]. A mixture of 20 mL of phosphoric and 180 mL of sulfuric acid (ACS reagent, Aldrich) was added to 1.5 g of graphite powder. Once a complete dispersion of the powder was obtained, 9 g of potassium permanganate (J.T. Baker, Mumbai, India) were mixed into the suspension. The mixture was kept under constant stirring for 330 min followed by the slow addition of 150 g of ice (de-ionized). The suspension was kept at 35 °C while 1.9 mL of 30% hydrogen peroxide were added. The supernatant and precipitate were separated by centrifugation. The precipitate, oxidized graphite (GO), was subject to three rinsing steps to remove undesired byproducts; the first one was conducted utilizing 30% HCl, the second with de-ionized water, and ethanol was used for the final step. The GO powder was then dried.

Doped graphene was produced *in situ* via the thermal reduction of graphite oxide in the presence of urea under inert atmosphere (reduction expansion synthesis). A sample in which no urea was employed, along mixtures with urea/GO ratios of 0.16, 0.2, 0.25, 0.33, 0.4, 0.6, 0.8, and 1 were used as precursors. The urea/GO mixtures were ground by hand in a mortar, placed in an alumina boat, and introduced into a quartz tube provided with airtight end adapters for atmosphere control. The quartz tube was flushed with nitrogen and inserted for 10 min into a Lindberg tubular furnace preheated to 800 °C. Previous studies demonstrated that the urea completely decomposes by 600 °C, however, by heating to 800 °C the mixtures seem to achieve a greater degree of expansion and oxygen groups loss, producing a higher quality product [28]. The resulting powders were characterized by multiple techniques as described below.

### 3.2. Sample Characterization

Scanning and transmission electron microscopy were employed to study the microstructure of the samples. A Zeiss Neon 40 (Carl Zeiss Inc., Thornwood, NY, USA) operating between 2–20 kV was employed for SEM studies. A JEOL 2010F FASTEM (JEOL Peabody, MA, USA) field emission gun STEM/TEM equipped with Gatan image filtering (GIF) (Gatan Inc., Warrendale, PA, USA) system was used to acquire the TEM and EELS data. Energy dispersive X-ray spectroscopy was performed utilizing the TEM. 

Powder samples were analyzed by X-ray diffraction using a Philips PW1830 X-ray diffractometer (Philips, Eindhoven, The Netherlands) operating in a continuous scan mode from a 2θ of 5° to 70°. Two second steps were utilized with a step size of 0.020° at a speed of 0.01° s^−1^. The Cu X rays generated had a λ of 1.5418 Å. The diffraction data was analyzed using Xpert Highscore Software (PANalytical, Westborough, MA, USA) using the ICDD PDF2 (International Center for Diffraction Data, Powder Diffraction Files version 2) database.

Raman spectroscopy studies were carried out using an inVia confocal raman microspectrometer (Renishaw Corp., Gloucestershire, UK) with 514-nm laser excitation under ambient conditions. Spectra were recorded using a total accumulation time of 20 s. 

Nitrogen isotherms were used to determine total surface area and micropore volume distribution employing Brunauer Emmet Teller (BET) and Horvath-Kawazoe (HK) methodologies, respectively. A Quantachrome Nova 4200e surface area and pore size analyzer (Quantachrome, Boynton Beach, FL, USA) using 9 mm large bulb long cell was employed for this analysis. Samples were degassed following an increasing temperature profile, starting at 100 °C for 30 min, followed by 300 °C for 150 min.

Thermogravimetric and evolved gas mass spectral analyses were performed to identify the various off gassing temperatures and constituents of heated samples. A Netzsch STA 449 F3 Jupiter connected to a MS403C Aëolos mass spectrometer (Netzsch GmbH & Co. KG Selb, Germany) was used. The temperature programmed oxidation of the doped samples generated in the furnace was conducted in an atmosphere that contained 80% inert gas and 20% oxygen (simulated air) with the intent of promoting the burn off of both the un-doped graphene and the doped samples.

### 3.3. Electrochemical Testing

Capacitor and battery electrodes were fabricated using a mixture of the as-produced graphene or doped graphene, acetylene black, *N*-Methyl-2-pyrrolidone (NMP) and polyvinylidene fluoride (PVDF) (MIT Corp., Richmond, CA, USA). For supercapacitor testing, the resultant slurry was spread onto 25 μm thick roughened nickel foil to a height of 350 μm using a film applicator, placed into a Barnstead Lab-Line L-C oven (Thermo Scientific, Waltham, MA, USA) at 100 °C for 1 h and then punched into 15 mm disks using a MTI Corporation precision disc cutter. The completed electrodes were assembled in pairs between two acrylic non-conductive polymeric blocks, separated by a celgrad membrane (Celgrad, Charlotte, NC, USA) soaked in electrolyte KOH solution. For battery electrode preparation, slurries were cast on a copper foil using a blade-applicator. The dried electrodes (thickness ~0.2 mm) were cut to 12 mm-diameter disks, transferred to an Ar-filled glovebox, and assembled in CR2032 coin cells in half-cell configuration (lithium metal anode). Electrochemical testing was conducted in an electrolyte containing 1M LiPF_6_ in EC:DMC:DEC (1:1:1). 

Battery and capacitor testing was conducted using a Maccor Model 4200M tester (Maccor, Tulsa, OK, USA). Capacitance was evaluated by applying three different current sources for a set number of cycles and measuring the resultant voltage trends. The currents and number of cycles utilized were 150, 350, and 500 μA for 50, 50, and 20 cycles, respectively. Battery testing was conducted using a charge/discharge rate current of C/10. 

## 4. Conclusions

We were able to successfully dope graphene with nitrogen functional groups with a high level of control over the degree of doping utilizing the reduction expansion synthesis (RES) method. 

SEM and TEM analysis revealed that the graphene produced resulted in thin sheets of disordered graphene, as is typical for samples made by reduction of graphite oxide. EELS analysis provided unequivocal evidence that nitrogen was embedded in the structure of graphene. Similarly, EDS shows nitrogen peaks in the doped samples further demonstrating the effectiveness of RES in doping the graphene. The BET surface area measurements for samples with diverse doping levels show an exponential decay as the amount of urea used in the precursor increase. These values can be controlled by modifying the amount of urea in the precursor samples.

XRD analysis of various levels of doped graphene revealed peak shifts indicating lower interplanar spacing “*d”* of the (002) planes as the concentration of nitrogen increased. The Raman intensity ratio between D and G band, *I*_D_*/I*_G_, increases with increasing urea content while the G band position remains nearly unchanged. These results confirmed the trends observed by BET analysis and indicated that there is a pronounced surface area loss as the amount of doping increases. 

All doped samples demonstrated to have high thermal stability up to 550 °C when studied by TGA and mass spectral analysis performed under temperature programmed oxidation conditions. The MS data corroborated that the amount of urea in the precursor samples can be directly linked to the amount of nitrogen species lost during temperature programmed oxidation/burn off of the samples, thus providing a path to control the amount of nitrogen species included in the structure and the surface area of the products.

Indeed, the introduction of nitrogen impurities by RES method in graphene produced from GO had a large effect in the graphene structure and improved the performance of electrodes based on such nitrogen doped graphene. Doping graphene with nitrogen clearly represents a step ahead in the development of electrode materials for new energy storage devices and the method presented herein provides a route to introduce a controlled amount of nitrogen and imprint a specific surface area in the products.

## Supplementary Materials

The following are available online at www.mdpi.com/1996-1944/8/10/5359/s1.
