# Supplementary material for: Reduction Expansion Synthesis as Strategy to Control Nitrogen Doping Level and Surface Area in Graphene
Source: Materials (Basel). 2015 Oct 16;8(10):7048–58. doi: 10.3390/ma8105359 (PMC5455405; doi:10.3390/ma8105359)
Supplement: Supplementary file 1 [file materials-08-05359-s001.pdf]

Supplementary Materials

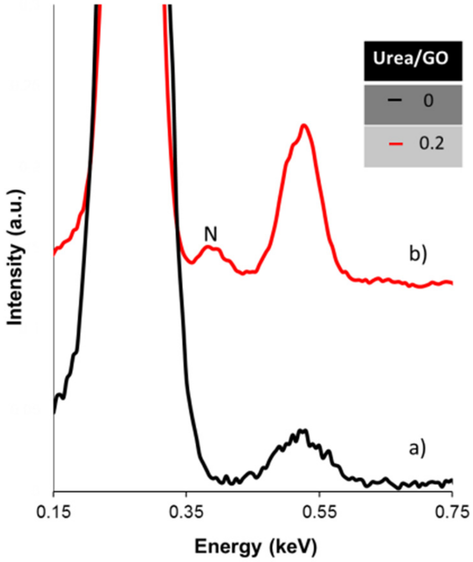

Figure S1. Energy dispersive X-ray spectroscopy (EDS) spectra of samples of reduced graphene. (**a**) Graphene from bare GO (no urea) and (**b**) Graphene doped from precursor with urea/GO ratio = 0.2 showing a small nitrogen peak (N-K peak at 392 eV).
